# Comparison of a Point-of-Care FilmArray Test to Standard-of-Care Microbiology Test in Diagnosis of Healthcare Associated Infections in a Tertiary Care Pediatric Intensive Care Unit

**DOI:** 10.3390/antibiotics11040453

**Published:** 2022-03-27

**Authors:** Ahmed A. El-Nawawy, Manal A. Antonios, Medhat E. Tawfik, Marwa A. Meheissen

**Affiliations:** 1 Department of Pediatrics, Faculty of Medicine, Alexandria University, Alexandria 21512, Egypt; dr_anawawy@yahoo.com (A.A.E.-N.); malakmanal@yahoo.com (M.A.A.); medhat2011fornow@gmail.com (M.E.T.); 2 Department of Medical Microbiology and Immunology, Faculty of Medicine, Alexandria University, Alexandria 21512, Egypt

**Keywords:** FilmArray, resistance genes, standard of care, culture, PCR

## Abstract

Background: Rapid and accurate identification of healthcare associated pathogens is crucial for early diagnosis and treatment of infections. This study aimed to assess the performance of a point-of-care multiplex polymerase chain reaction (PCR) in diagnosis of pathogens and their antibiotic resistance genes in bloodstream infections, pneumonia and meningitis/encephalitis in a pediatric intensive care unit (PICU). Methods: A retrospective cross-sectional study was conducted on pediatric patients diagnosed with healthcare associated infections at Alexandria University PICU, Egypt. A total of 111 samples from 98 patients were subjected simultaneously to standard-of-care microbiology testing (SOCMT) and molecular testing by BioFire multiplex PCR. Results: In comparison to SOCMT, the BioFire FilmArray^®^ had a better diagnostic yield with broncho-alveolar lavage (BAL) (45 vs. 21) and cerebrospinal fluid (CSF) samples (five vs. none) (*p* ≤ 0.0001). *Klebsiella pneumoniae* was the most common pathogen in BAL (*n* = 19 by BioFire, *n* = 9 by SOCMT) and blood (*n* = 7, by SOCMT and BioFire) samples, while *Streptococcus pneumoniae* was the most common in CSF samples. BioFire showed 95.8% overall percent agreement, 100% positive percent agreement and 95.6% negative percent agreement with SOCMT. All phenotypically confirmed resistant isolates had resistance genes by the BioFire FilmArray^®^ (100%). The turnaround time (TAT) of positive results by the FilmArray panels was 1–1.5 h in comparison to 48–72 h by SOCMT (*p* ≤ 0.001). Conclusions: The results of the current study confirm the utility of the BioFire FilmArray^®^ in making early decisions regarding patients’ diagnosis and management of infection in the PICU, in terms of rapid TAT and appropriate antimicrobial use.

## 1. Introduction

Microbiological diagnosis of pneumonia, bloodstream infection (BSI), and meningoencephalitis in pediatric intensive care patients is often problematic, due to difficulty in detection of the causative pathogen by routine microbiological methods, especially if the patient is already on antimicrobial therapy [1,2]. Additionally, a large proportion of pediatric infections are known to be of viral etiology and their diagnosis needs specific tests [1]. Moreover, the predictive value of markers that differentiate bacterial and viral infection is low and not yet standardized [3].

Microbiological cultures are still the routine standard of care methods for diagnosis of different types of infections, especially in developing countries. Cultures have the advantage of isolation of the pathogen with guidance of appropriate antimicrobial therapy according to susceptibility results. However, recovery of pathogens may be problematic due to previous antibiotic exposure prior to sample collection in intensive care unit (ICU) patients. Moreover, the turnaround time of most cultures is variable, from 48–72 h [2,4]. Additionally, supplementary non-routine tests for diagnosis of atypical bacteria or viral pathogens are usually needed in the pediatric population [4]. As a result of the lack of timely routine diagnostic methods, there is often extensive abuse of antibiotics, antivirals and antifungals in the management of healthcare associated infections (HAIs) in ICUs [5].

The introduction of polymerase chain reaction (PCR) based methods in diagnosis of infections in ICUs allowed the generation of rapid results within hours of sample collection. With the advent of molecular diagnostics, syndromic-based multiplex molecular assays have been introduced for the diagnosis of respiratory, bloodstream, and central nervous system (CNS) infections [6]. The possibility of introduction of these tests as point-of-care testing methods in ICUs has dramatically changed the diagnosis of infectious diseases. These tests offer rapid detection of the causative pathogens coupled with detection of antimicrobial resistance gene markers two to three days before routine culture results. This will eventually limit unnecessary broad-spectrum antibiotic use. Furthermore, directed antiviral treatment will be possible for pediatric respiratory and meningoencephalitis cases [5,7].

This study aimed to assess the performance of a point-of-care multiplex PCR (FilmArray^®^; BioFire Diagnostics, BioMérieux, Salt Lake City, UT, USA) in diagnosis of causative agents as well as of the bacterial antibiotic resistance genes in the bloodstream, lower respiratory and CNS infections, in comparison to standard-of-care microbiology tests (SOCMT), in an attempt to improve the turnaround time for diagnosis and to enhance antimicrobial treatment protocols at a university-affiliated Pediatric Intensive Care Unit (PICU).

## 2. Patients and Methods

### 2.1. Study Setting and Design

This retrospective cross-sectional study included all patients with clinically suspected infections, aged one month to 14 years, who were admitted from June 2019 till June 2020 in the PICU, Alexandria University Hospitals, Egypt. The study was in accordance with the ethical standards of the institutional and national research committee and with the 1964 Helsinki Declaration. The Institutional Review Board of Alexandria University, Faculty of Medicine, approved this study (IRB No: 00012098, FWA No: 00018699). A statement of informed consent was taken from all subjects or their guardians before inclusion in the study.

**Figure 1:**
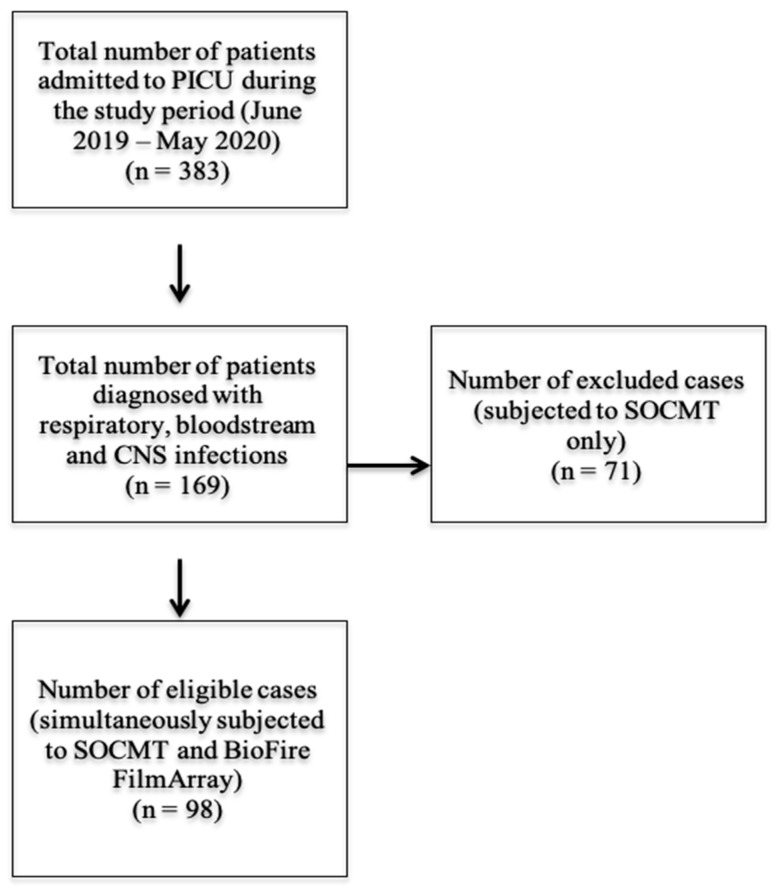
Schematic representation of sample eligibility in the present study.

Patients diagnosed with bloodstream, respiratory or CNS infections [8,9] who were subjected simultaneously to SOCMT and molecular testing by BioFire multiplex PCR assay were included in the study (Figure 1).

### 2.2. Patients’ Data

Demographic and clinical data such as age, gender, underlying disease, risk factors, severity of illness (by pediatric mortality and prognostic scores (pediatric index of mortality; PIM2 and pediatric logistic organ dysfunction; PELOD), antimicrobial regimens were collected from the PICU computerized filling system (Nawawy’s Egyptian Program for Pediatric Intensive Care Unit; licensed under patent restoration number 003073).

### 2.3. Standard of Care Microbiology Testing (SOCMT)

Bacterial and fungal cultures were performed as part of SOCMT in the microbiology laboratory according to standard operating procedures. Blood cultures were processed in the BACT/ALERT 3D system (BioMérieux, Durham, NC, USA). Cerebrospinal fluid (CSF) samples were processed after centrifugation. Respiratory samples (broncho-alveolar lavage (BAL, mini-BAL) were processed using a semi-quantitative culture method. All samples as well as positive signal blood culture bottles were cultured on chocolate agar, blood agar, MacConkey’s and Sabouraud dextrose agar. All culture plates were examined for growth after 24 and 48 h. Any growth from blood and CSF samples was considered positive. Cultures were considered positive for a respiratory pathogen if ≥10^4^ CFU/mL were detected. Isolated bacterial or yeast colonies were identified using standard biochemical tests (e.g., catalase, coagulase, oxidase, triple sugar iron, citrate, urease, motility, ornithine and lysine decarboxylation, germ tube test) [10]. Antimicrobial susceptibility testing was performed by disc diffusion and/or broth microdilution according to CLSI guidelines [11] and as per lab SOPs. Screening for antimicrobial resistance was performed as recommended by CLSI for the specific pathogen (cefoxitin 30 μg disc diffusion for methicillin resistance in staphylococci, combined disc diffusion test for ESBL, and carbapenem inactivation method for carbapenemases in Gram-negative bacilli). All cultures’ data was drawn from the university microbiology laboratory information system.

Detection of atypical bacteria (Chlamydia, Mycoplasma, Legionella) as well as the detection of viral respiratory pathogens is not included in SOCMT in the microbiology laboratory. Therefore, only data regarding bacterial and fungal etiology were available.

### 2.4. BioFire FilmArray^®^

The BioFire system was introduced in the PICU since June 2019 as a point-of-care test. It is a multiplex PCR combined with array detection using an automated closed system that isolates, amplifies and detects nucleic acid for multiple causative pathogens within a single specimen in one step (FilmArray, BioFire Diagnostics, BioMérieux, Salt Lake City, UT, USA, Serial Number 2FA06414). The possible organisms and the corresponding bacterial resistance genes are identified within 60–90 min from admission and management plans are designed accordingly.

The BioFire Pneumonia (PN) panel *plus* is intended for the simultaneous detection of 27 respiratory viral and bacterial nucleic acids, as well as select antimicrobial resistance genes (*CTX-M*, *NDM*, *IMP*, *OXA-48-like*, *KPC*, *VIM*, *mecA/C* and *MREJ*). It has the advantage of reporting bacteria semi-quantitatively (10^4^, 10^5^, 10^6^, or ≥10^7^ copies/mL) [4,7], while the blood culture identification (BCID) panel provides results for 24 different BSI organisms and organism groups and three antimicrobial resistance genetic markers (*mecA*, *vanA/B*, *KPC*) [12]. The Meningitis/Encephalitis (ME) panel is capable of the simultaneous detection of fourteen bacteria, viruses and yeasts that can cause CNS infections [13].

The FilmArray PN panel *plus*, BCID, and ME panels were processed according to the manufacturer’s instructions. Briefly, 100 μL of the positive blood culture fluid was diluted with sample dilution, 200 μL of CSF sample, and 200 μL of respiratory sample, captured with the sample swab and mixed in dilution buffer, then was loaded in the designed pouches and inserted into the FilmArray instrument.

All data generated from the instrument were reported to the clinicians in real time and recorded in Microsoft Excel sheets. All data regarding the results of the BioFire PN *plus*, BCID and ME panels were collected retrospectively as part of this study.

### 2.5. Comparison between the Results of SOCMT and BioFire FilmArray

Comparison between the results of SOCMT and the BioFire panels was done regarding the types of pathogens identified, the antimicrobial resistance pattern and the turnaround time (TAT) from ordering the test to final release of results.

### 2.6. Evaluation of the Potential Impact of BioFire Results on Antimicrobial Therapy

Potential impacts of the BioFire results on antibiotic therapy were evaluated and classified as: (a) Stop antimicrobial (antibiotic, antiviral, antifungal) if the results of BioFire excluded bacterial/viral/fungal etiology; (b) start antiviral if results of BioFire confirmed viral etiology and antiviral was not already included in empiric therapy; (c) de-escalation of antimicrobial if a narrower spectrum antibiotic was described (e.g., carbapenem or colistin was replaced by cephalosporins); (d) broaden antimicrobial spectrum if a shift to a more broad spectrum antibiotic was done (e.g., cephalosporin was replaced by carbapenem or colistin) and (e) no change if the empiric antimicrobial therapy was not modified after BioFire results.

### 2.7. Statistical Analysis

Data was analyzed using the statistical package for the Social Sciences (SPSS), version 25 (IBM Corp., Armonk, NY, USA). Quantitative data was expressed as mean and standard deviation. Qualitative data was described as number and percentage. A chi-square test or Fisher Exact test were performed to test the association between the BioFire FimArray and SOCMT positive samples, as appropriate. Additionally, *p*-values ≤ 0.05 were considered statistically significant. Total TAT was calculated in hours and was compared using a two-sample *t*-test. BioFiore performance was evaluated by percent agreement (positive, negative, overall) between the BioFire and SOCMT results.

## 3. Results

### 3.1. Patients’ and Samples’ Clinical Characteristics

Ninety-eight patients, admitted to the PICU during the study period, were diagnosed with respiratory, blood stream or central nervous system infection, with a mean age of 22.9 ± 35.6 months, and male: female ratio of 1.17:1. Their mean PIM-2 and PLEOD scores were 35.01 ± 28.13 and 4.01 ± 1.60, respectively.

Out of the 98 patients, 61 patients (62.2%) were diagnosed with ventilator-associated pneumonia (VAP), 19 (19.4%) with meningitis/meningoencephalitis and 14 (14.3%) with BSI. Three patients (3.1%) had combined VAP and BSI, while one patient (1%) had meningitis, BSI and VAP. Thus, a total of 65 VAP, 20 meningitis/meningoencephalitis and 18 BSI cases were included in the study. A total of 111 samples (72 miniBAL, 23 CSF, 16 blood) from the 98 patients were analyzed by both SOCMT and the BioFire FilmArray.

### 3.2. Diagnostic Performance of SOCMT and BioFire FilmArray

In the current study, in comparison to SOCMT, the BioFire FilmArray had a better diagnostic yield with mini-BAL and CSF samples. The PN panel identified 45 positive cases (62.5%), in comparison to 21 cases (29.2%) by culture. Therefore, the panel detected an additional 33.3% of VAP cases (*p* < 0.0001). The ME Panel also identified five cases (21.7%) that were totally missed by culture. Regarding blood samples, both the BCID Panel and SOCMT identified all pathogens, except in one case where Neisseria meningitidis was detected by BioFire only.

Overall, 54 mini-BAL and 11 blood organisms were identified by the BioFire panels, in comparison to 23 mini-BAL and ten blood by SOCMT. All CSF organisms were only identified by the ME Panel. The most frequent pathogens identified by both methods are mentioned in Table 1 and Table 2.

Klebsiella pneumoniae was the most common pathogen in mini-BAL (n = 19 by BioFire, n = 9 by SOCMT) and blood (n = 7, by SOCMT and BioFire) samples, while Streptococcus pneumoniae was the most common in CSF samples (n = 4 by BioFire, n = 0 by SOCMT). Although comparison between the results of the SOCMT and the FilmArray PN panel plus did not show significant differences with all pathogens, the FilmArray succeeded in identifying fastidious pathogens, such as Hemophilus influenzae (*p* = 0.051) and Streptococcus pneumoniae (*p* =0.170), and to increase the yield of other pathogens, such as Klebsiella pneumoniae (*p* = 0.742), Pseudomonas aeruginosa (*p* = 0.250) and MRSA (*p* = 1.000) (Table 3).

The copy number of bacterial targets identified by the FilmArray PN panel plus is mentioned in Table 4. Copies/mL are slightly higher when the organism is also isolated from culture. As the current study is a retrospective one, we could not retrieve data on bacterial count in mini-BAL cultures, as the lab report is only released if growth is ≥10^4^ CFU/mL.

Since testing for viral pathogens is not included in SOCMT of our hospital, viral pathogens were only detected by the BioFire FilmArray. At least one viral pathogen was detected by the PN panel in 66.6% (48/72) of mini-BAL samples. Out of 72 mini-BAL samples, 21 (29.2%) were viral, 18 (25%) were bacterial and 27 (37.5%) were of combined viral/bacterial etiology. Rhinovirus/Enterovirus followed by Respiratory Syncytial Virus were the most commonly identified viruses by the PN panel (29.2%, 26.4% respectively) (Table 5). None of the meningoencephalitis cases was positive for viral etiology by the ME panel.

Percent agreement was calculated to assess the overall performance of the FilmArray panels for detection of bacterial and fungal targets. BioFire showed 95.8% overall percent agreement, 100% positive percent agreement, and 95.6% negative percent agreement with SOCMT (Table 6).

### 3.3. Screening for Antimicrobial Resistance

The results of phenotypic antimicrobial resistance revealed nine Klebsiella spp. and three Pseudomonas spp. ESBL producers, 13 Klebsiella spp., three Pseudomonas spp. and three Acinetobacter spp. carbapenemase producers. All phenotypically confirmed resistant isolates had resistance genes by the BioFire FilmArray (100%). All ESBL producers had the bla_CTX-M_ gene. Carbapenemase producers had positive bla_NDM_ (*n* = 12), bla_OXA-48_ (*n* = 4), bla_VIM_ (*n* = 3), and bla_KPC_ (*n* = 1) genes. Additionally, the mini-BAL methicillin resistant Staph. aureus case was positive for the mecA gene by the FilmArray.

### 3.4. Turn-Around Time (TAT)

The TAT of positive results (organism identification and antimicrobial resistance detection) by the FilmArray BCID and PN panels was 1.5 h in comparison to 48–72 h by SOCMT (*p* ≤ 0.001). Likewise, the TAT of positive results by the FilmArray ME panel was one hour compared to 72 h by SOCMT (*p* ≤ 0.001). Furthermore, the TAT of negative results by the FilmArray BCID panel (1.5 h) was significantly faster than that of SOCMT (5 days) and of the FilmArray PN (1.5 h), and the ME (1 h) panels were shorter than SOCMT (48–72 h) (*p* ≤ 0.001).

### 3.5. Potential Impacts of BioFire Results on Antimicrobial Therapy

The results of BioFire allowed the stoppage of antimicrobial therapy in about 50% of patients (38/98; 38.8% stop antibiotics, 7/98; 7.1% stop antifungal, 2/98; 2% stop antiviral). In an additional six patients (6.1%) de-escalation was performed. Twenty-six patients (26.5%) were subjected to broadening of antimicrobial spectrum treatment, and 12 patients (12.2%) started antiviral treatment. On the other hand, no change in the antimicrobial protocols was needed in 37.8% of patients. Multiple impacts could have been possible for one patient. All these impacts, except for antiviral treatment, could have been applied in the light of the SOCMT culture results, but BioFire offered significantly earlier results for intervention, which would definitely affect the patients’ outcome.

## 4. Discussion

To the best of our knowledge, this study is the first to evaluate the performance of the BioFire FilmArray as a point-of-care molecular test in the PICU in Egypt.

The use of the PN panel *plus* in the current work resulted in a 33.3% increase in specimens identified as positive as compared to SOCMT methods. These specimens were reported as “No growth” or “Mixed growth of normal flora of upper respiratory tract in a count of <10^4^” in SOCMT. The use of the PN panel in the study of Buchan et al. [4] and Ozongwu et al. [14] resulted in a larger percentage (63.3% and 63.4% respectively) of positive samples. The interpretation of positive BioFire PN panel results and their clinical significance remains an unresolved issue in culture negative cases. Nevertheless, Rand et al. [15] mentioned that they validated the results of extra targets identified by BioFire that were missed by culture using another PCR method, and they confirmed the true positivity of the results. Buchan et al. [4] stated that the results of (culture negative-PN panel positive at 10^4^ copies/mL) cases should be considered with caution in the context of patients’ clinical and other laboratory results, as they were traditionally non-reported based on the cutoff value of routine culture methods (10^4^ CFU/mL). They also concluded that if laboratories provide the semiquantitative values of the BioFire PN panel in their report, they should warn the clinician that these values will be greater than the corresponding quantitative culture results [4]. On the other hand, in a multicenter evaluation of the BioFire PN panel from lower respiratory tract samples (846 BAL and 836 sputum), many false positive results were recovered (mainly for *Staphylococcus aureus*, *Hemophilus influenzae*, *Moraxella catarrhalis*, *Pseudomonas aeruginosa*) with a specificity of 87.2% compared to culture results [7]. False positive results could lead to unnecessary antibiotic therapy.

In our study, the PN panel identified at least one viral pathogen in 66.6% of mini-BAL samples. Similarly, Human rhinovirus/enterovirus was the most frequently detected in a previous study [4]. The lack of a SOCMT viral test in the current work prevents a comprehensive comparison of the PN panel’s performance in detecting viral targets. However, the considerably high rate of respiratory viral pathogens detected in the current study highlights the need to include viral testing in routine PICU microbiology diagnostic work-up.

Regarding blood samples, since the BCID panel is already designed to process positive Signal blood cultures, both SOCMT and the BCID Panel successfully identified all blood culture samples. Only one culture-negative positive Signal blood culture was identified as *Neisseria meningitidis* by BioFire. As the patient was not manifesting signs of meningitis or meningococcemia, the result of the BioFire test was considered a false positive, and the Signal blood culture was also considered a false positive. Concerning the ME panel, the five positive cases were missed by culture. Thus, antibiotics should not be stopped in children with a high suspicion of meningitis based on a negative culture result, especially if there is CSF laboratory evidence of bacterial infection (neutrophils). The small number of blood and CSF samples in the current work hindered the comparison of our results to previous studies. Nevertheless, out of 145 infants, the FilmArray ME panel resulted in two false positives cases in the study of Blaschke et al. [16]. *Streptococcus pneumoniae* was positive in one case with no CNS clinical manifestations. *Cryptococcus* was detected in a second case that was diagnosed by another type of infection (urinary tract infection).

It is important to mention that we did not run any additional tests to validate the accuracy of the BioFire panel findings in culture-negative specimens. However, the detection of both viable and non-viable organisms by PCR, as well as detection of low-abundance targets and those not recovered in culture due to their fastidious nature, in addition to the antibiotic abuse, had resulted in an increase in positive results reported by BioFire.

The overall percent agreement between all the panels used in the current study and SOCMT was very high (95.8%), with 100% PPA and 95.6% NPA for bacterial and yeast targets. Similarly, Buchan et al. [4] reported 96.2% PPA and 98.1% NPA for bacterial targets in the PN panel. Webber et al. [17] also found a high overall percent agreement (99.2%) between the PN panel and culture.

The identification of antimicrobial resistance genes by the BioFire panels has several advantages. Previous studies observed that detection of resistance genes by molecular methods has been associated with better patient outcomes in terms of length of hospital stay and mortality rate [18,19]. In addition, many healthcare organizations have particular contact isolation procedures in place for patients who have resistant bacteria with these markers [4]. On the other hand, the presence of resistance genes does not confirm phenotypic resistance, as non-expression could be the case. Furthermore, their absence should not be interpreted as a susceptible phenotype, since antibiotic resistance could be mediated by several genes not included in the test.

The TAT of all panels used in this study was significantly shorter than the SOCMT TAT, ranging from 1–1.5 h, and this was the actual TAT, since the BioFire FilmArray is a point-of-care test in the PICU; no time was needed for sample transport or reporting of results by the laboratory. In their study, Blaschke et al. [16] reported a BioFire TAT of about 3 h, considering sample transport and laboratory reporting.

One of the main goals of this research was to evaluate how the results of BioFire may affect antimicrobial therapy. Notably, antimicrobial therapy for 37.8% of patients have not been changed as a result of BioFire results; nevertheless, early confirmation that these patients were receiving optimal treatment aided in care management. Moreover, the fact that 50% of patients have stopped antimicrobial therapy and 12.2% have started antivirals according to the results of BioFire emphasizes the importance of raising the awareness of intensivists about the predominance of pediatric viral infections in the PICU and demonstrates the urgent need to standardize the PICU empiric antimicrobial treatment protocols.

The present study has several limitations: it was a single center, so generalization of the results is not applicable. The date of the BioFire analysis and the change in antimicrobial therapy were approximately matched, making the results of BioFire’s impact on antimicrobial therapy liable to inaccuracy. Additionally, it is unclear whether or not other factors influenced clinical judgments on whether or not to start, stop, or continue antibiotic therapy. Moreover, the true impact of the BioFire FilmArray and subsequently modifications in antimicrobial therapy on quality metrics including length of ICU stay, mortality rate, as well as on healthcare resource utilization will have to be determined through prospective randomized controlled trials rather than retrospective analysis.

## 5. Conclusions

The results of the current study confirm the utility of the BioFire FilmArray in making early decisions regarding patients’ diagnosis and management of infection in the PICU, in terms of rapid TAT and appropriate antimicrobial use. The study also highlights the importance of the BioFire FilmArray in diagnosis of viral infections and difficult-to-culture bacteria. Importantly, the BioFire FilmArray is a supplement to early decision-making, not a substitute for clinical evaluation and consideration of additional laboratory results.

## Figures and Tables

**Table 1:** Distribution of pathogens isolated by standard of care microbiology testing (SOCMT).

SOCMT (*n* = 111)	No.	%
miniBAL culture (*n* = 72)	72	100
No growth	51	70.8
*Klebsiella* spp. *	7	9.7
*Pseudomonas* spp. *	6	8.3
*Acinetobacter* spp. *	5	7.0
*Klebsiella* spp., *pseudomonas* spp. *	2	2.8
*Staphylococcus aureus* (MRSA) ^	1	1.4
Total # of positive cases	21	29.2
Blood culture (*n* = 16)	16	100
No growth	6	37.5
*Klebsiella* spp. *	7	43.8
*E. coli* *	1	6.2
*Acinetobacter* spp. *	1	6.2
*Candida albicans* #	1	6.2
Total # of positive cases	10	62.5
CSF culture (*n* = 23)	23	100
No growth	23	100

*: Gram-negative bacteria, ^: Gram-positive bacteria, #: Fungus.

**Table 2:** Distribution of bacterial and fungal targets identified by the BioFire FilmArray.

BioFire FimArray Results	No.	%
FilmArray^®^ Blood Culture Identification (BCID) Panel (*n* = 16)		
Negative	5	31.3
*Klebsiella pneumoniae* *	7	43.8
*E. coli* *	1	6.3
*Acinetobacter baumannii* *	1	6.3
*Neisseria meningitidis* *	1	6.3
*Candida albicans * #	1	6.3
Total number of positive cases	11	68.7
FilmArray^®^ Pneumonia panel *plus* (*n* = 72)		
Negative (*n* = 6) and viral etiology (*n* = 21)	27	37.5
*Klebsiella pneumoniae* *	15	20.8
*Pseudomonas aeuroginosa* *	7	9.7
*Haemophilus influenzae* *	6	8.3
*Acinetobacter calcoaceticus-baumannii complex* *	4	5.6
*Streptococcus pneumoniae* ^	4	5.6
*Klebsiella pneumoniae/Pseudomonas aeruginosa* *	3	4.2
*Staphylococcus aureus* ^	1	1.4
*Hemophilus influenzae* */*Staphylococcus aureus* ^	1	1.4
*Stahylococcus aureus* ^/*Streptoccocus.pneumoniae* ^/*Hemophilus influenzae* *	1	1.4
*Pseudomonas aeruginosa*/*Hemophilus influenzae* *	1	1.4
*Klebsiella pneumoniae* */*Streptococcus pneumoniae* ^	1	1.4
*Acinetobacter calcoaceticus-baumannii complex*/*Pseudomonas aeruginosa* *	1	1.4
Total number of positive cases	45	62.5
Total number of identified bacterial organisms	54	
FilmArray^®^ Meningitis/Encephalitis (ME) Panel (*n* = 23)		
Negative	18	78.3
*Streptococcus pneumoniae* ^	4	17.4
*Hemophilus influenzae* *	1	4.3
Total number of positive cases	5	21.7

*: Gram-negative bacteria, ^: Gram-positive bacteria, #: Fungus.

**Table 3:** Comparison between the results of the BioFire FimArray and SOCMT for the positive samples.

Organism	Number of Pathogens Identified				*p* Value
	Blood Culture (*n* = 10)		FilmArray^®^ Blood Culture Identification (BCID) Panel (*n* = 11)		
	No.	%	No.	%	
*Klebsiella pneumoniae*	7	70.0	7	63.6	1.000
*E. coli*	1	10.0	1	9.1	1.000
*Acinetobacter calcoaceticus-baumannii complex*	1	10.0	1	9.1	1.000
*Candida albicans*	1	10.0	1	9.1	1.000
*Neisseria meningitidis*	0	0.0	1	9.1	1.000
	miniBAL culture (*n* = 23)		FilmArray^®^ Pneumonia Panel *plus* (*n* = 54)		
	No.	%	No.	%	
*Klebsiella pneumoniae*	9	39.1	19	35.2	0.742
*Pseudomonas aeruginosa*	8	34.8	12	22.2	0.250
*Acinetobacter calcoaceticus-baumannii complex*	5	21.7	5	9.3	0.154
*Staphylococcus aureus* (MRSA)	1	4.3	3	5.6	1.000
*Haemophilus influenzae*	0	0.0	9	16.7	0.051
*Streptococcus pneumoniae*	0	0.0	6	11.1	0.170
	CSF culture (*n* = 0)		FilmArray^®^ Meningitis/Encephalitis (ME) Panel (*n* = 5)		
	No.	%	No.	%	
*Streptococcus pneumoniae*	0	0.0	4	80.0	-
*Hemophilus influenzae*	0	0.0	1	20.0	-

**Table 4:** Comparison between the distribution of bacterial copy number identified by the BioFire FilmArray Pneumonia panel *plus* in culture positive and negative cases for the specific type of the organism.

Organism	BioFire FilmArray Pneumonia Panel *plus* Copy Number (Copies/mL)			
	10^4^	10^5^	10^6^	≥10^7^
*Klebsiella pneumoniae* (*n* = 19)				
Culture positive (*n* = 9)	-	1	6	2
Culture negative (*n* = 10)	2	1	4	3
*Pseudomonas aeruginosa* (*n* = 12)				
Culture positive (*n* = 8)	1	2	1	4
Culture negative (*n* = 4)	-	2	1	1
*Acinetobacter* spp. (*n* = 5)				
Culture positive (*n* = 5)	2	1	-	2
Culture negative (*n* = 0)	-	-	-	-
*Staphylococcus aureus* (MRSA) (*n* = 3)				
Culture positive (*n* = 1)	-	-	1	-
Culture negative (*n* = 2)	-	2	-	-
*Haemophilus influenzae* (*n* = 9)				
Culture positive (*n* = 0)	-	-	-	-
Culture negative (*n* = 9)	1	-	3	5
*Streptococcus pneumoniae* (*n* = 6)				
Culture positive (*n* = 0)	-	-	-	-
Culture negative (*n* = 6)	2	-	4	-

**Table 5:** The distribution of viruses identified by the FilmArray^®^ Pneumonia panel *plus*.

Viruses	Number of miniBAL Specimens	%
Human Rhinovirus/Enterovirus	21	29.2
Respiratory Syncytial Virus	19	26.4
Influenza A	8	11.1
Adenovirus	7	9.7
Parainfluenza Virus	5	6.9
Coronavirus	2	2.7
Human Metapneumovirus	1	1.4
Influenza B	1	1.4
MERS-CoV	0	0
None detected	8	11.1
Total	72	100

MERS-CoV: Middle East Respiratory Syndrome Coronavirus.

**Table 6:** Percent agreement between SOCMT and the BioFire FimArray for the detection of bacterial and fungal targets. (*n* = 98).

Bacterial/ Fungal Targets	Number of Samples				PPA (95% CI)	NPA (95% CI)	OPA (95% CI)
	SOCMT Positive/BioFire Positive	SOCMT Positive/BioFire Negative	SOCMT Negative/BioFire Positive	SOCMT Negative/BioFire Negative			
*Klebsiella pneumoniae*	16	0	10	72	100 (80.6–100.0)	87.8 (79.0–93.2)	89.8 (82.2–94.4)
*E. coli*	1	0	0	97	100 (20.7–100.0)	100 (96.2–100.0)	100 (96.2–100.0)
*Pseudomonas aeruginosa*	8	0	4	86	100 (67.6–100.0)	95.6 (89.1–98.3)	95.9 (90.0–98.4)
*Acinetobacter* spp.	6	0	0	92	100 (61.0–100.0)	100 (96.0–100.0)	100 (96.2–100.0)
*Haemophilus influenzae*	0	0	10	88	-	89.8 (82.2–94.4)	89.8 (82.2–94.4)
*Neisseria meningitidis*	0	0	1	97	-	99.0 (94.4–99.8)	99.0 (94.4–99.8)
*Streptococcus pneumoniae*	0	0	10	88	-	89.8 (82.2–94.4)	89.8 (82.2–94.4)
*Staphylococcus aureus*	1	0	2	95	100 (20.7–100.0)	97.9 (92.8–99.4)	98.0 (92.9–99.4)
*Candida albicans*	1	0	0	97	100 (20.7–100.0)	100 (96.2–100.0)	100 (96.2–100.0)
Total	33	0	37	812	100 (89.6–100.0)	95.6 (94.1–96.8)	95.8 (94.3–96.9)

SOCMT: Standard-of-care microbiology testing; PPA: positive percent agreement; NPA: Negative percent agreement; OPA: Overall percent agreement; CI: Confidence interval.

## Data Availability

Not applicable.
